# Measuring serious violence: a comparison of self-reported and police-recorded outcomes in a UK birth cohort linked to local police data.

**DOI:** 10.23889/ijpds.v7i3.1876

**Published:** 2022-08-25

**Authors:** Rosie Cornish, Alison Teyhan, John Macleod, Kate Tilling, Iain Brennan

**Affiliations:** 1 University of Bristol; 2 University of Hull

## Objectives

- To compare self-reported and police-recorded serious violence using data from a longitudinal UK birth cohort linked to local (Avon and Somerset Constabulary) police records
- To assess the risk of police-recorded serious violence according to study participation status across the lifecourse

## Approach

We linked data from the Avon Longitudinal Study of Parents and Children (ALSPAC) to local police records on charges, cautions and other out of court disposals. We compared the risk of self-reported serious violence at 8 time points from 15 to 25 years to police-recorded serious violence at these ages. We then compared the risk of police-recorded serious violence among those actively participating in ALSPAC to those not participating at various time points from pregnancy onwards. We used logistic regression to examine whether differences in risk could be explained by socio-demographic and family characteristics associated with ALSPAC participation.

## Results

The sample included 12,662 participants who had received fair processing materials and had not opted out of linkage to police records. They linked to a total of 6,283 offences, of which 933 were classified as serious violence (involving a total of 530 individuals). Self-reported serious violence in the past year was particularly high at 15 years (23.5%); at other ages it ranged from 3.7% (22 years) to 8.7% (20 years). Police-recorded serious violence was lower at all ages, peaking at 1.0% at 17 and 18 years. The risk of police-recorded serious violence was higher among those not participating in ALSPAC than among active participants, particularly during adolescence and early adulthood. This difference was only partially explained by socio-demographic and other factors.

## Conclusion

A key advantage of linkage to police records is it enables outcomes to be measured irrespective of study participation status as relying only on active participants leads to bias. Combining self-reported and police-recorded outcomes allows us to derive measures of offending that take account of the biases inherent in each.

